# "Concomitant immunity" in murine tumours of non-detectable immunogenicity.

**DOI:** 10.1038/bjc.1985.6

**Published:** 1985-01

**Authors:** R. A. Ruggiero, O. D. Bustuoabad, R. D. Bonfil, R. P. Meiss, C. D. Pasqualini

## Abstract

**Images:**


Br. J. Cancer (1985), 51, 37–48

### "Concomitant immunity" in murine tumours of non-detectable immunogenicity

R.A. Ruggiero, O.D. Bustuoabad, R.D. Bonfil, R.P. Meiss & C.D. Pasqualini

*Instituto de Investigaciones Hematológicas and Centro de Estudios Oncológicos, Academia Nacional de Medicina, Las Heras 3092, 1425 Buenos Aires, Argentina.*

**Summary** Various immunization assays were used to demonstrate the lack of immunogenicity of three BALB/c tumours of spontaneous origin and of a fourth one resulting from foreign body tumorigenesis. All four tumours inhibited the growth of a second implant of the same tumour into the contralateral flank. In our tumour models "concomitant immunity" (1) was not mediated by macrophage or T-cell dependent immune reactions: both thymectomized BALB/c and nude mice (treated or untreated with silica) gave the same results as intact mice; (2) showed some degree of non-specificity, inhibiting the growth of a different tumour in 3/4 cases; though, the existence of a specific component could not be discarded; (3) was proportional to the volume of the primary tumour at the time of the second challenge; (4) was dependent on actively growing primary tumour, not being obtained with progressively increasing daily inocula of irradiated tumour cells; (5) was detectable in an actively growing secondary tumour: recurrent growth after partial surgical excision was inhibited and (6) involved cytostasis of the secondary tumour: a syngeneic graft of the overlying skin led to tumour growth while histological studies revealed the presence of viable tumour cells. It is postulated that "concomitant immunity" or resistance can be generated without the active participation of the immune system and that tumour-related factors are, in certain cases, responsible for blocking the growth of secondary tumours.

In cancer research there is a phenomenon known as "concomitant immunity" according to which a tumour-bearing host resists a second implant of its own tumour at a different site. It was first described by Ehrlich (1906) and Bashford (1908) devised the term. Since then, apart from a few isolated papers (for reviews see Roffo, 1914; Woglom, 1929; Vaage, 1971) this paradoxical phenomenon remained forgotten for almost 60 years. Even now only few laboratories are dedicated to the study of "concomitant immunity" (for review, see Gorelick, 1983*b*) in spite of its possible relevance to the mechanism of metastasis control. In this regard, it has been repeatedly observed that the removal of a murine metastasizing tumour is followed by an abrupt increase in metastatic growth (Crile & Deodhar, 1971; Gorelik, 1982). This would suggest that the primary tumour exerted a controlling action on its metastases which could be considered as a natural "secondary implant". On the whole, "concomitant immunity" has been evaluated during the growth of tumours induced by carcinogenic agents which are strongly immunogenic and in consequence an immunological interpretation has been favoured. However, the demonstration of non-specificity in some systems (Kearney & Nelson, 1973; North & Kirstein, 1977) and the detection of "concomitant immunity" with tumours of weak antigenicity (Yuhas *et al.*, 1975; Malenica & Milas, 1979) do not fall in easily with this explanation. Furthermore, the phenomenon has also been observed in association with human tumours (Southam & Brunschwig, 1961; Southam, 1968) which can best be compared with animal tumours of spontaneous origin. In this paper, the detection of "concomitant immunity" is reported with three mouse tumours of spontaneous origin and with a fourth one induced by foreign body tumorigenesis; the non-immunogenic nature of these tumours is demonstrated by various procedures and in consequence the immunological interpretation of the phenomenon is questioned.

Correspondence: R.A. Ruggiero, Sección Leucemia Experimental, Instituto de Investigaciones Hematológicas, Academia Nacional de Medicina, Las Heras 3092, 1425 Buenos Aires, Argentina.
Received 3 July 1984; and in revised form, 31 August 1984.

### Materials and methods

### *Animals*

BALB/c, and F1 (BALB/c × DBA/2) mice of both sexes and 2–4 months old were used throughout. They were raised in our own colony and maintained on Cargill pellets and water *ad libitum*. *Nude* BALB/c mice were obtained from the Comisión Nacional de Energía Atómica, Argentina, and kept under relatively aseptic conditions.

© The Macmillan Press Ltd., 1985

Animals were age and sex matched within each experiment.

*Tumours*

The following 4 tumours were used for "concomitant immunity" studies.

*LB* Lymphoid leukaemia which arose spontaneously in a 6 month old BALB/c male. It was maintained by s.c. serial passages in syngeneic mice and was used between passages 40 and 87. It grows to a large size *in situ* and at autopsy, infiltration of lymph nodes, spleen and liver can be seen. The number of s.c. injected viable tumour cells required to give a 50% probability of lethality ($LD_{50}$) was $\sim 10^3$. In order to determine whether active viral replication was involved, one month old BALB/c mice ($n=11$) were inoculated i.p. with acellular extracts of LB: they did not develop leukaemia, during 15 months of observation.

*CEI* Undifferentiated epidermoid carcinoma which arose spontaneously in a 12 month old BALB/c female; it was maintained by syngeneic s.c. serial passages and used between passages 2–6. It is a slow growing tumour with $LD_{50}$ of 11,250 cells and at autopsy lung metastases are occasionally encountered.

*CM* Mammary adenocarcinoma which arose spontaneously in a 12 month old BALB/c female; it was maintained by syngeneic s.c. serial passages and used between passages 1–3. It is a slow growing tumour with $LD_{50}$ of $10^4$ cells which does not give rise to metastases.

*PX* Fibrosarcoma which was induced by a foreign body (glass cylinder) 6 months after s.c. implantation in a BALB/c female, as described previously (Pasqualini *et al.*, 1973). It was maintained by syngeneic s.c. serial passages of which passages 106–135 were used. The tumour grows *in situ* and does not give rise to metastases.

The following 4 tumours were used as controls in different experiments:

*P-388* Lymphoid leukaemia induced by methylcholanthrene in 1957; subline Ps78R07 was recently obtained from Arthur D. Little Co., Cambridge, Mass. It was maintained by i.p. serial passages in (BALB/c x DBA/2) F1 mice but used as a s.c. transplant.

*CS* A mammary carcinoma originally induced by MMTV (murine mammary tumour virus) kindly supplied by Dr Diana Lopez, University of Miami. The tumour was transplanted s.c. in BALB/c mice. It is slow growing with $LD_{50}$ of $3.2 \times 10^4$ cells and does not metastasize.

*MC-C* Fibrosarcoma which arose in a 5 month old BALB/c male 3 months after the implantation of a methylcholanthrene pellet. It was maintained as a syngeneic serial line of which passages 3–5 were used. It is a slow growing tumour with a regression rate of 13% and $LD_{50}$ of $5 \times 10^4$ cells.

*MC-D* Fibrosarcoma induced by methylcholanthrene in a 6 month old BALB/c male which appeared 4 months after the implantation of a methylcholanthrene pellet; it has a regression rate of 44% and $LD_{50}$ of $10^6$ cells. It was used as s.e. transplant between passages 3–5.

*Thymectomy in newborn mice*

Within 24 h after birth BALB/c mice were anaesthetized on ice and their thymus was removed by vacuum aspiration. Thymectomy was controlled at autopsy by macro and microscopic observations. Histological observations showed reductions in number and size of lymph node follicles with atrophy of the paracortical zone (thymus-dependent).

*Experimental model for "concomitant immunity"*

The primary tumour was implanted s.c. in the right flank of mice followed at different intervals by a second s.c. implantation in the contralateral flank of either the same tumour or a different one; suppression or delay of growth of the second tumour implant was considered as a measure of "concomitant immunity". Tumour volume was expressed according to the formula of Attia & Weiss (1966): volume $= 0.4\ (ab^2)$ where a and b represent the larger and smaller diameters respectively. Excellent reproducibility was found from one experiment to another and therefore data from several experiments were pooled.

*Immunization assays*

The following procedures were carried out:

*Irradiated cells* Cell suspensions were irradiated with 90 Gy in a plastic irradiation chamber; X-rays were generated in a Philips 250/15 Radiotherapy apparatus at 220 kv, 14 mA and filtered with 1 mm Al. The dose rate was $3.51\ Gy\ min^{-1}$ at a focus-target distance of 29 cm. In most cases the animals were pretreated with 2 s.c. doses of $4 \times 10^6$ irradiated tumour cells, 15–22 and 7–12 days before tumour challenge. In one case, $200\ mg\ kg^{-1}$ of cyclophosphamide (Endoxan, Labinca S.A. Argentina) was inoculated i.p. 2 days before pretreatment. In another experiment, only one dose of $2 \times 10^6$ irradiated cells in complete Freund's adjuvant was inoculated, 15 days before tumour challenge.

*Mitomycin C-treated cells* Tumour cell suspensions ($10^7 ml^{-1}$) were incubated at 37°C for 45 min with $50 \mu g ml^{-1}$ of Mitomycin C (Sigma Chemical Co., St Louis, USA) and washed 3 times with saline solution. The animals were pretreated s.c. with $10^6$ of these cells 15 days before tumour challenge.

*Glutaraldehyde-treated cells* Tumour fragments were fixed in 3% glutaraldehyde (Sigma Chemical Co., St Louis, USA) in PBS at 37°C for 45 min, washed 3 times at 4°C, and implanted s.c. 14 and 8 days before tumour challenge.

*Heat-treated cells* Tumour cells ($10^6$–$10^7$) heated at 80–90°C for 1 min were inoculated s.c. 19 and 7 days before tumour challenge.

*Cold-treated cells* Tumour cells ($2 \times 10^6$) which had been maintained at −15°C for 45 min were inoculated s.c. 21 and 7 days before tumour challenge.

*Sublethal doses* Mice which had survived a first tumour implant were re-inoculated with various doses of cell suspensions of the same tumour.

*Tumour implantation and excision* S.c. tumour implants were surgically excised when their volume had reached 400–600 mm³; 2–3 weeks later, a second tumour implant was carried out in the contralateral flank in the mice which had not relapsed.

### *Tumour neutralization test*

The anti-tumour activity of lymph node or spleen cells of tumour-bearing mice was investigated with the *in vivo* Winn test (Winn, 1961), by mixing them with tumour target cells at various lymphocyte-target cell ratios. The cells were inoculated by the s.c. route and tumour growth evaluated.

### *Statistical analysis*

$\chi^2$ and Student's *t*-test were used. Differences were considered significant when the *P* value was $\leq 0.05$.

### Results

### *Detection of "concomitant immunity"*

*"Concomitant immunity" against LB*: BALB/c mice were inoculated s.c. with $10^6$ LB cells (average latency to death, 22 days) in the right flank; they received a second s.c. implant of either $10^6$, $10^5$ or $10^4$ LB cells in the left flank 0, 3, 6, 9 and 11 days later when the primary tumour volumes were 0, 0, 150, 400 and 800 mm³, respectively. The controls received only the left flank inoculum. As can be seen from Table I, "concomitant immunity" was proportional to the tumour growth of the primary implant. Thus, mice with a primary tumour implant of $10^6$ cells resisted a simultaneous secondary implant of $10^4$ LB cells but not of higher doses. Similarly, a second implant of $10^5$ LB cells carried out on day 6 did not grow while one of $10^6$ led only to a slight decrease in second tumour takes. Finally, all the animals challenged with a second implant of $10^6$ cells on day 9 or 11, were able to resist it. In two other experiments, using primary implants of $10^5$ and $10^4$ LB cells, (average latency of 24 and 30 days respectively) similar results were obtained, except that "concomitant immunity" was detected later. In all cases the observation period between the inoculum in the left flank and the death of the animals was 11–22 days.

*"Concomitant immunity" against CEI*: A total of 20 BALB/c mice were inoculated s.c. with $5 \times 10^5$ CEI cells in the right flank (average latency, 50 days); when the tumour size reached 40 mm³ ($n=6$), 300 mm³ ($n=9$) or 1800 mm³ ($n=5$), a second implant of $5 \times 10^5$ CEI cells was carried out in the left flank. A control group of 18 mice received only the tumour cells in the left flank. As can be seen in Figure 1, no "concomitant immunity" was detected in animals bearing the smallest primary tumour but was evident in the other two groups bearing larger primary tumours in which only 3/9 and 3/5, respectively, showed tumour growth at the end of the experiment.

*"Concomitant immunity" against CM*: Seven BALB/c mice received a primary s.c. implant of $2 \times 10^6$ CM cells in the right flank (average latency, 50 days); when tumour size reached 600 mm³ ($n=4$) or 2000 mm³ ($n=3$), a second s.c. implant of $1.5 \times 10^6$ CM cells was carried out in the contralateral flank. The controls ($n=13$) received only the second tumour challenge. As shown in Figure 2, in the presence of the larger primary tumour, the second implant did not grow at all while in the other group the secondary implant showed significantly retarded growth. The observation period between the inoculum in the left flank and the death of the animals was 25–33 days.

*"Concomitant immunity" against Px*: A total of 46 BALB/c mice were implanted s.c. with a solid

**Table I** Inhibition of tumour growth at the site of the second tumour implant as a measure of "concomitant immunity" generated by a lymphoid leukaemia (LB) in normal, thymectomized and *nude* BALB/c mice

| *Tumour inocula* | | *Exp.*[a] | *Control* | *Exp.* | *Control* | *Exp.* | *Control* |
|---|---|---|---|---|---|---|---|
| 1st (right flank) | | | | | | | |
| No. of LB cells (day 0) | | $10^6$ | — | $10^6$ | — | $10^6$ | — |
| 2nd (left flank) | | | | | | | |
| No. LB cells (on day) | ↓ | $10^6$ | $10^6$ | $10^5$ | $10^5$ | $10^4$ | $10^4$ |
| | | *Tumour takes in left flank/No. of mice* | | | | | |
| *Normal mice* | | | | | | | |
| | (0) | 12/12 | 6/6 | 6/6 | 6/6 | 1/12[d] | 12/12 |
| | (3) | 7/7 | 3/3 | 5/5 | 4/4 | | |
| | (6) | 14/18 | 12/12 | 0/36[d] | 18/18 | | |
| | (9) | 0/6[c] | 4/4 | | | | |
| | (11) | 0/6[c] | 6/6 | | | | |
| *xT mice*[b] | | | | | | | |
| | (6) | | | 0/6[c] | 6/6 | | |
| | (9) | 0/6[c] | 6/6 | | | | |
| *nude mice* | | | | | | | |
| | (7) | | | 1/10[d] | 7/7 | | |
| | (11) | 1/6[c] | 6/6 | | | | |

[a]Experimental group received both LB tumour inocula while the control group received only the second inoculum.
[b]BALB/c mice thymectomized at birth.
[c]$P<0.01$ as calculated by $\chi^2$.
[d]$P<0.001$.

**Figure 1** Resistance of BALB/c tumour-bearing mice to a second challenge of the same tumour ($5\times10^5$ CEI cells) carried out when the first implant had reached a volume of 40 mm³ (○——○), 300 mm³ (▲——▲) or 1800 mm³ (△——△); controls (●——●) received only the second tumour challenge. The secondary tumour grew in (○——○) as in the controls, while it was significantly decreased (*t*-test) in both incidence and volume (mm³ ± s.e.) in the other groups.

**Figure 2** Resistance of BALB/c tumour-bearing mice to a second challenge of the same tumour ($1.5\times10^6$ CM cells) carried out when the first implant had reached a volume of 600 mm³ (○——○) or 2000 mm³ (▲——▲); controls received only the second tumour challenge (●——●). Secondary tumour growth (mm³ ± s.e.) was significantly decreased (*t*-test) as compared with the controls, in direct relation with primary tumour volume.

**Figure 3** Resistance of BALB/c tumour-bearing mice to a second challenge of the same tumour (1 mm³ Px fragment) carried out when the first implant had reached a volume of 600 mm³ (○——○), 1400 mm³ (△——△), 2700 mm³ (▲——▲) or 5400 mm³ (□——□); controls (●——●) received only the second tumour challenge. Secondary tumour growth (mm³ ± s.e.) was significantly decreased (*t*-test) in direct relation with primary tumour volume except in the case of the largest primary tumour which did not lead to "concomitant immunity" (□——□).

**Figure 4** Non-specific resistance of (BALB/c × DBA/2) F1 mice bearing LB tumour to a second challenge of P-388 tumour cells. *Experimental groups*: F1 mice were inoculated on day 0 with $10^6$ LB tumour cells in the right flank; then they received in the contralateral flank $10^5$ P-388 tumour cells on day 7 (△——△) or $10^6$ P-388 tumour cells on day 9 (○——○). *Control groups*: Received only $10^5$ (▲——▲) or $10^6$ (●——●) P-388 tumour cells in the left flank. A significant decrease (*t*-test) in P-388 tumour growth (expressed as mm³ ± s.e.) was obtained in mice bearing LB tumour as compared with the corresponding controls.

fragment (1 mm³) of Px by trocar, in the right flank (average latency, 30 days) and when tumour size reached 600 mm³ ($n=12$), 1400 mm³ ($n=12$), 2700 mm³ ($n=12$) or 5400 mm³ ($n=10$), a secondary s.c. implant of Px was carried out in the contralateral flank. The controls ($n=28$) received only the second tumour challenge. As can be seen in Figure 3, the growth of the second implant was significantly inhibited in direct proportion to the size of the primary tumours except in the presence of a very large one, in which case, the second implant reached a volume similar to that of the controls.

It is important to point out that in the four models studied the primary tumour grew and killed the host independently of the fate of the second inoculum.

*Nonspecificity of "concomitant immunity"*

Animals bearing a primary tumour were challenged with a second implant of a different tumour, to test the specificity of "concomitant immunity".

*In a first experiment (LB-P388)* sixteen (BALB/c × DBA/2) F1 mice were inoculated, on day 0, with $10^6$ LB cells in the right flank and were divided into 2 groups, 10 mice receiving on day 7, $10^5$ P-388 cells in the left flank and 6 mice receiving on day 9, $10^6$ P-388 cells. The 16 control mice received only the corresponding inoculation in the left flank, that is, only $10^5$ or $10^6$ P-388 cells. As shown in Figure 4, a marked and significant decrease in the growth of the second implant was observed in both experimental groups, as compared with the controls.

*In a second experiment (CS-LB)* six BALB/c mice were inoculated s.c., on day 0, with $10^6$ CS cells (slow growing tumour); 15 days later, when this tumour was not yet palpable, a second s.c. implant of $10^6$ LB cells (fast growing tumour) was carried out in the contralateral flank. In this case "concomitant immunity" was evident against CS which, on day 35, did not grow in 3 animals while in the remaining 3 it grew less well (600 mm³) than in the 6 controls (970 mm³) bearing only CS. The phenomenon observed was due to the rapid growth of the second tumour implant (LB) which caused "concomitant immunity" against the primary tumour (CS). The observation period was limited to

35 days since by that time the animals began to die of LB tumour.

*In a third experiment* (*CS-LB*) four BALB/c mice received $10^6$ CS cells in the right flank and 49 days later, when the tumour measured 6000 $mm^3$, $10^6$ LB cells were inoculated s.c. in the contralateral flank. Within 2 weeks the 4 mice had died of the CS tumour, during which time the LB implant had not grown at all in 2 mice while in the remaining 2 it had grown much less (300 $mm^3$) than in the corresponding 6 controls (1600 $mm^3$).

*In a fourth experiment* (*LB-Px*) twelve BALB/c mice were s.c. implanted with $10^6$ LB cells; then the animals received 6 or 9 days later a solid fragment (1 $mm^3$) of Px cells in the contralateral flank. No difference in tumour growth of the second implant was observed as compared with mice bearing only a Px implant i.e., no "concomitant immunity" was detected in this case.

*"Concomitant immunity" in athymic mice*

The participation of T lymphocytes in the development of "concomitant immunity" was studied in both *nude* mice and in mice thymectomized within 24 h after birth. Results are summarized in Table I.

A total of 12 BALB/c mice thymectomized at birth were inoculated s.c. with $10^6$ LB cells when they were 2–3 months old; on day 6, when the tumour measured 200 $mm^3$, 6 mice received a second implant of $10^5$ LB cells in the contralateral flank, while the remaining 6 animals received $10^6$ LB cells s.c. on day 9, when the primary tumour had reached 1000 $mm^3$. As can be seen in Table I, "concomitant immunity" was observed in both groups as compared to controls bearing only the second implant and did not differ from that registered in euthymic mice. In another experiment, 16 BALB/c *nude* mice were inoculated s.c. with $10^6$ LB cells and on day 7, when the tumour measured 120 $mm^3$, 10 of them received s.c. $10^5$ LB cells in the contralateral flank, while the remaining 6 received s.c. on day 11, a second implant of $10^6$ LB cells. As shown in Table I, "concomitant immunity" was evident in both groups as compared with controls bearing only the second implant and did not differ from that registered in euthymic mice. It is interesting to note that in *nude* mice LB cells grew more slowly than in normal mice; therefore the second implant was carried out 1 and 2 days later.

*"Concomitant immunity" in silica treated mice*

Since silica treatment is considered to depress the functions of macrophages (Allison *et al.*, 1966; Gorelick, 1983*a*) and presumably of NK cells (Djeu *et al.*, 1979; Gorelik, 1983*a*), in order to elucidate their participation in the development of "concomitant immunity", 6 *nude* and 6 euthymic BALB/c mice received a s.c. implant of $10^6$ LB cells in the right flank and 7 days later were inoculated i.v. with 2 mg of silica (1 $\mu$m particles, Sigma Chemical Co., St Louis, USA) and 1 h later they were challenged s.c. with $10^5$ LB cells in the left flank. The development of "concomitant immunity" was not altered by this treatment, i.e. there was no secondary tumour growth. The same results were obtained in 3 BALB/c mice which received 1 mg of silica s.c. at the same site and simultaneously with the secondary tumour implant i.e. "concomitant immunity" was again evident.

The effect of i.v. silica (2 mg) on NK cells and macrophages was evaluated in preliminary experiments.

*Effect on NK cells* Splenocytes of 2 BALB/c mice treated 3 days before with i.v. silica showed a 14% decrease in cytolysis (using a 4 h $^{51}Cr$ release assay) of YAC-1 cells (a tissue culture cell line of YAC, a Moloney virus-induced lymphoma of A/J origin), as compared with splenocytes of 2 normal BALB/c mice.

*Effect on macrophages* The phagocytic activity (K) of the reticuloendothelial system was studied by measuring the capacity of test animals to clear i.v. injected colloidal carbon from their peripheral blood and was determined by the formula:

$$K = \frac{\log_{10} OD_3 \min - \log_{10} OD_{15} \min}{12 \min},$$

where $OD_3$ min and $OD_{15}$ min represent the optic densities of the blood, 3 and 15 min after the carbon injection (Biozzi *et al.*, 1953; Levy & Wheelock, 1975). Phagocytic activity of 6 BALB/c mice treated 1 day before with i.v. silica was significantly depressed ($K = 0.038 \pm 0.008$) as compared with that of 5 normal mice ($0.080 \pm 0.005$; $P < 0.01$).

*Undetectable immunogenicity of the four tumours generating "concomitant immunity"*

*Immunization assays: LB* As can be seen in Table II, different procedures were carried out. In all cases, 12–30 putatively immunized BALB/c mice together with the corresponding normal controls were challenged s.c. with $10^6$, $10^5$, $10^4$, $10^3$ or $10^2$ LB cells and the $LD_{50}$ value was calculated. In general, the immunization procedures were not capable of increasing $LD_{50}$ or altering tumour growth. A slight increase in $LD_{50}$ was registered in

**Table II** Lack of immunogenicity of a lymphoid leukaemia (LB) expressed as $LD_{50}$ in syngeneic BALB/c mice pretreated with different immunization procedures, using two methylcholanthrene-induced tumours (MC-C and MC-D) as immunogenic controls

| | $LD_{50}$ | | |
|---|---|---|---|
| *Immunization assays* | *LB* | *MC-C* | *MC-D* |
| Control | | | |
| (without pretreatment) | $10^3$ | $5\times10^4$ | $10^6$ |
| 1 Implantation-excision | —[a] | $>4\times10^6$ | $>5\times10^6$ |
| 2 Sublethal tumour doses | $10^3$ | | $>5\times10^6$ |
| 3 X-irradiated cells + complete Freund adj. | $5.6\times10^3$ | | |
| 4 X-irradiated cells + cyclophosphamide | $1.3\times10^3$ | | |
| 5 X-irradiated cells | $7.7\times10^2$ | $>5\times10^6$ | |
| 6 Mitomycin C treated cells | $7\times10^2$ | | |
| 7 Cold-inactivated cells | $5.6\times10^3$ | | |
| 8 Heat-inactivated cells | $\leqslant3\times10^3$ | | |
| (% spontaneous regression) | 0 | 13 | 44 |

[a]The mice died of leukaemic dissemination except for 2 which did not resist a subsequent tumour challenge.

mice treated with irradiated cells in complete Freund's adjuvant or with cold-inactivated cells, however, when these presumably immunized mice were re-challenged with graded doses of LB cells, no difference in $LD_{50}$ was observed compared with the controls. Furthermore, when LB cells fixed in glutaraldehyde were used to immunize 12 BALB/c mice a subsequent s.c. tumour challenge with $10^6$ LB cells grew at the same rate as in the controls; this group could not be included in Table II because only 1 dose was used as challenge so that $LD_{50}$ could not be calculated. Another procedure used to test tumour immunogenicity was that of tumour implantation followed by surgical extirpation. However, out of a total of 78 mice bearing a 6–12 day LB growth resulting from the s.c. inoculation of $10^5$ or $10^6$ cells, only 2 survived and proved to be non-immunized since a subsequent challenge with $10^4$ LB cells grew as in the controls. The remaining mice died of leukaemic dissemination in spleen, lymph nodes and liver and a few of them showed local relapse at the extirpation site. As can be seen in Table II, the lack of immunogenicity of LB is in sharp contrast with the results obtained with 2 methylcholanthrene-induced fibrosarcomas (MC-C and MC-D). The spontaneous regression rate of these methylcholanthrene tumours is a further index of their strong immunogenicity contrasting with the fact that mice bearing LB never showed tumour regression in more than 500 animals used in these experiments.

*CEI* The only immunization procedure carried out was that of tumour implantation and excision. The results indicate that in tumour-excised mice, $LD_{50}$ (3200 cells) was even lower than that observed in the controls (11,250 cells). Out of a total of 30 mice operated upon, 15 relapsed locally. Spontaneous tumour regression was never seen (100 mice).

*CM* Of 16 mice bearing a CM tumour which were operated upon, the 12 animals which did not show local recurrence were challenged s.c. with graded doses of CM cells. Both the pretreated and control mice showed the same $LD_{50}$ of $10^4$ cells and similar latency to death, ranging from 50 to 65 days depending on the doses used. No spontaneous tumour regression was ever seen (100 mice).

*Px* Two immunization assays, pretreatment with sublethal tumour doses and implantation-excision were carried out in 52 BALB/c mice. Challenge doses consisted of fragments of Px of different size and the results obtained did not reveal any degree of resistance (measured as tumour takes or growth rate) in pretreated or operated mice as compared with the controls. $LD_{50}$ was not calculated because of the difficulty of counting viable fibrosarcoma Px cells in a reproducible way. Local recurrence in excised-tumour mice was 50%. No spontaneous tumour regression was ever seen (300 mice).

*Winn test* Spleen or lymph node cells were obtained from BALB/c mice bearing either one or two LB implants, (of which the second implant failed to grow because of "concomitant immunity") and mixed with $5\times10^3$ LB cells in different proportions. The cell mixture was then inoculated s.c. into syngeneic mice. As shown in Table III, no significant difference ($\chi^2$ test) could be observed in the experimental groups compared with controls receiving only LB cells mixed with normal spleen or lymph node cells.

*Miscellaneous observations concerning the mechanism responsible for "concomitant immunity"*

Taking into account that the development of "concomitant immunity" was proportional to the tumour volume of the first implant, the possibility was considered that the repeated inoculation of irradiated LB cells in progressively larger daily doses, always at the same s.c. site, might mimic tumour load and thereby generate "concomitant

**Table III** *Winn test*: Tumour incidence after inoculation of different lymphocyte: LB-tumour-cell ratios in BALB/c mice.

| *Lymphocytes from* | *Ratio lymphocytes:LB cells* | *Tumours n/total* | % |
|---|---|---|---|
| | 0:1 $(5\times10^3)$ | 15/30 | 50 |
| Normal spleen | 50:1 | 8/12 | 66 |
| | 100:1 | 5/12 | 42 |
| | 10:1 | 2/6 | 33 |
| Normal lymph node | 50:1 | 11/18 | 61 |
| | 100:1 | 6/12 | 50 |
| Spleen of LB-bearing mice | 50:1 | 6/6 | 100 |
| (1 implant[a]) | 100:1 | 2/6 | 33 |
| Spleen of LB-bearing mice | 50:1 | 10/12 | 83 |
| (2 implants[b]) | 100:1 | 5/6 | 83 |
| Right inguinal lymph node of | 50:1 | 2/6 | 33 |
| LB-bearing mice (1 implant[a]) | 100:1 | 6/6 | 100 |
| Right inguinal lymph node of | 50:1 | 6/6 | 100 |
| LB-bearing mice (2 implants[b]) | 100:1 | 6/6 | 100 |
| Left inguinal lymph node of | 75:1 | 6/6 | 100 |
| LB-bearing mice (1 implant[a]) | | | |
| Left inguinal lymph node of | 10:1 | 1/6 | 16 |
| LB-bearing mice (2 implants[b]) | 50:1 | 8/12 | 66 |
| | 100:1 | 6/12 | 50 |
| Left inguinal lymph node of | 50:1 | 6/6 | 100 |
| LB-bearing *nude* mice (2 implants[b]) | 75:1 | 6/6 | 100 |

[a]The first implant was always carried out s.c. in the right flank.
[b]The second implant of LB cells in the left flank was not growing due to "concomitant immunity."

immunity". Therefore, 7 BALB/c mice were inoculated s.c. into the right flank with 9 daily doses imitating the increase in volume of a tumour originated from an inoculum of $10^6$ LB cells; on day 6, the animals were challenged s.c. with $10^5$ viable LB cells in the left flank. No decrease, rather a slight increase in tumour size, was observed compared with control mice bearing only the tumour implant in the left flank; i.e. no "concomitant immunity" had been generated.

In order to determine whether "concomitant immunity" could inhibit not only secondary implants but also actively growing tumour cells, 18 BALB/c mice were inoculated s.c. with $10^6$ LB cells in both flanks. On day 6, the incipient LB tumour growing in the left flank of 12 mice was partially excised, leaving behind a mass of $2\times10^5$ or $10^4$ LB cells, while in the remaining 6 mice, the tumour was excised on day 9, leaving behind a cluster of $\sim2\times10^3$ LB cells. The number of LB cells left behind was calculated indirectly in 12 mice which were similarly operated upon and sacrificed to count the remaining tumour cells. The fate of the LB cells left at the operation site in the presence of the growing tumour in the opposite flank was compared with that of controls operated upon in the same way, but not bearing an LB tumour in the right flank. The results showed that $2\times10^5$ LB remaining cells grew independently of the presence of the tumour in the contralateral flank, while $10^4$ cells grew but at a slightly lower rate than in the corresponding controls. However, when the number of remaining LB cells was around $2\times10^3$ and the size of the tumour at the contralateral flank was larger, only 1/6 animals showed tumour growth, while in 5/6 controls the tumour grew; the observation period between the operation and the death of the animals was 8–14 days and it is interesting to note that $2\times10^5$, $10^4$ or $2\times10^3$ LB growing cells left behind after excision gave rise to a palpable tumour in only 2, 3 and 4 days respectively. This is a very short tumour latency compared with that required for s.c. inocula of $2\times10^5$, $10^4$ or $2\times10^3$ LB cells: 7, 14 and 18 days, respectively.

In order to determine whether viable tumour cells were present in the lymph nodes draining a second LB implant, which was not growing because of "concomitant immunity", the following experiment was carried out. Two BALB/c *nude* mice bearing a growing LB tumour in the right flank and a second LB implant in the left flank, were sacrificed; the left axillary and the left inguinal lymph nodes were excised. These 4 nodes were implanted s.c. by trocar in the left flank of 4 BALB/c mice, 2 of which had received in the right flank a s.c. LB implant of $10^6$ cells, 7 days before. The results indicate that as a first implant, these lymph node cells led to tumour growth, but as a second implant they did not grow, demonstrating that the lymph nodes draining non-growing secondary implants contained viable tumour cells which could be inhibited by "concomitant immunity".

Such a cytostatic mechanism was confirmed by a syngeneic skin graft. Donor skin from 8 BALB/c mice came from the site of a second LB implant which had been carried out 7 or 11 days before and which was not growing. In all of 8 grafted mice a tumour developed at the site of the graft and grew progressively, leading to the death of the animals. This would indicate that "concomitant immunity" was capable of blocking tumour growth by a reversible cytostatic mechanism. As preliminary results, a further confirmation of the cytostatic state of the tumour cells undergoing "concomitant immunity" was obtained from histological studies. (Figure 5).

### Discussion

"Concomitant immunity" was demonstrated in association with the four tumours studied; three of these arose spontaneously in our mouse colony and the fourth resulted from the implantation of a foreign body; all of them proved to be non-immunogenic by several immunological procedures. The intensity of "concomitant immunity" was proportional to the tumour volume of the first implant. However, with foreign body tumorigenesis the phenomenon tended to disappear when the first implant had reached a critical size; such a large volume was never attained with the other three tumours which may explain why a terminal decrease in "concomitant immunity" was not seen, although particular differences among tumours in the generation of this phenomenon cannot be discarded.

Three different theories have been proposed to explain "concomitant immunity". Historically, the theory of atrepsis was the first one put forward by Ehrlich (1906). It postulates that nutrients essential for tumour growth are consumed by the primary tumour making it difficult for a second implant to develop. In our experiments, the fact that when a secondary implant of $10^5$ LB cells did not grow, one of $10^6$ could grow, does not seem to favour this explanation since the lack of nutrients should have inhibited both inocula, unless the larger one was able to attract more of the necessary potential growth factors. Furthermore, when fibrosarcoma Px had acquired a very large size and presumably should have been consuming more nutrients according to this theory, "concomitant immunity" was decreased.

The second theory was originally proposed by Bashford (1908) and states that as a tumour grows it generates an immunological response which even though it is not strong enough to inhibit primary tumour growth, is still capable of preventing the development of a relatively smaller second implant. In the last 15 years, this immunological explanation has had many advocates (Deckers *et al.,* 1973; Vaage, 1971; North *et al.,* 1982) but their experiments involved immunogenic tumours induced either by carcinogenic agents or by oncogenic viruses. Such an immunological mediation does not provide a satisfactory explanation for our experiments. Firstly, it has not been possible to demonstrate any degree of immunogenicity in the tumours studied. A similar lack of immunogenicity of tumours of spontaneous origin has been demonstrated by Hewitt *et al.* (1976) in 27 mouse tumours and by Middle & Embleton (1981) in 32 rat tumours. In the second place, *nude* mice or those thymectomized at birth showed the same development of "concomitant immunity" as the normal controls, which would presumably discount a main role for T-lymphocyte mechanisms. Moreover, the i.v. treatment of athymic and normal mice with silica did not alter the development of "concomitant immunity" which would not favour an active participation of macrophages. As for NK cells, in spite of a reduction in cytolysis of YAC-1 cells (14%) in i.v. silica treated mice, the small number of animals tested does not make it possible to discount their participation in the development of "concomitant immunity": further experiments are being carried out. Gorelik (1983*a*) working with long-passaged tumours also observed "concomitant immunity" in *nude* and in normal mice treated i.p. with silica.

The third theory was recently formulated by Gorelik *et al.* (1981) who suggested that tumour cells of the first graft produce or induce inhibitory factor(s) which suppress the replication of tumour cells of the second inoculum. This inhibitory factor(s) is held to be non-tumour specific and to function independently of the components of the immune system. The production of such a factor(s) would explain the suppressive effect which the local tumour exerts on its metastases as well as their accelerated growth following removal of the primary tumour mass. This theory could also explain the development of "concomitant immunity" in immunodeficient mice as well as its induction by non-immunogenic tumours. However, such antimitotic factors capable of inhibiting a

**Figure 5** *Experimental group* (a and b): Six days after a first s.c. implant of $10^6$ LB cells in the right flank, 12 BALB/c mice received a second s.c. implant of $10^5$ LB cells in the left flank which did not grow because of "concomitant immunity". Skin from this site was removed 7 days later and histologically analyzed. Note that, at the s.c. site of the second tumour implantation, there are non-infiltrating neoplastic LB cells (5a, H & E ×100 orig. mag.) which are morphologically well preserved with no signs of necrosis (5b, H & E ×250 orig. mag.). *Control group* (c): 12 BALB/c mice received only $10^5$ cells in the left flank; skin from this site was removed 7 days later and histologically analyzed. Note the presence of abundant neoplastic LB cells infiltrating the muscular layer and the dermis (5c, H & E ×100 orig. mag.). This tumour was macroscopically detectable.

second implant should presumably also affect the growth of the primary tumour since the same cells are involved. In our experiments, LB and Px tumours have gone through several passages without showing a decrease in their capacity to generate "concomitant immunity". It is not easy to visualize, in the context of this theory, why clones of tumour cells sensitive to this antimitotic factor(s) would be retained throughout the different passages escaping an elimination by natural selection.

It is possible that "concomitant immunity" could have two different causes: one immunological, detected only with immunogenic tumours and a second one, non-immunological, common to both immunogenic and non-immunogenic tumours: in the latter case, concomitant resistance would be a better denomination. The fact that North & Kirstein (1977) observed that in T-cell deprived mice, "concomitant immunity" generated by immunogenic tumours substantially decreased but *did not* disappear would favour such an interpretation.

An analysis of the three theories proposed to explain "concomitant immunity" reveals that the mechanisms implicated in its generation are not yet well understood; the data presented herein indicate that "concomitant immunity" or resistance generated by spontaneous non-immunogenic murine tumours would have the following characteristics: (1) It does not seem to be mediated by macrophage or T-cell dependent immune reaction; (2) It has some degree of non specificity: a significant growth inhibition of the secondary implant was obtained in 3/4 different tumour combinations; however, the fact that "concomitant immunity" observed in LB tumour-bearing mice against a second implant of LB was stronger than that observed against P-388 or CS indicates that a specific component (not necessarily immunological in our tumour models) cannot be discarded; (3) It would operate by a cytostatic mechanism: the second tumour implant remains viable as demonstrated by *in vivo* transplants and by histological studies. Using long-passaged tumours, Gorelik (1983*a*) arrived at similar conclusions. However, the questions we are now trying to address are how long do the cytostatic tumour cells of the secondary implant remain in this dormant state and whether they will eventually die; (4) Its induction is dependent on a growing tumour mass: it could not be generated with a serial and progressively higher inoculum of irradiated LB tumour cells mimicking the increasing tumour mass; (5) It affects not only secondary tumour grafts but also actively growing tumour cells: recurrent tumour growth after partial surgical excision could be inhibited by "concomitant immunity".

As a final consideration it must be noted that the detection of "concomitant immunity" presents several methodological difficulties. In effect, the long latency period of relatively small secondary implants may signify that the animals can die of the primary tumour before the secondary one has had a change to appear. On the other hand, a very large secondary implant can overcome "concomitant immunity" precluding its observation. Middle & Embleton (1981) working with 5 spontaneous rat tumours of non-detectable immunogenicity observed that only one of them led to "concomitant immunity"; however, with the other four tumours the second implant was carried out when the first one was either slightly or not palpable at all. When we did experiments in the same conditions "concomitant immunity" was not detected; a larger primary tumour, or else a proportionally smaller second inoculum was needed.

It can be argued that the non-proliferation of the secondary tumour due to "concomitant immunity" is comparable to dormant metastases. On the other hand, animal tumours of spontaneous origin have been considered as the most appropriate model for human cancer (Hewitt, 1978). Therefore, experiments designed to explain the mechanisms involved in "concomitant immunity" with these tumours may eventually be of benefit for the control of human metastases.

The authors are grateful to Drs O. Stutman, M.M. Bracco, M.A. Isturiz and I.M. Piazzon for critical review of the manuscript, and to Messrs J.J. Portaluppi and A. Morales for excellent technical assistance. We thank Drs S.L. Rabasa and S. Olabuenaga for constructive suggestions and Dr M. Giordano for drawing the figures. This study was supported by grants from CONICET (Consejo Nacional de Investigaciones Científicas y Técnicas) and FUNDALEU (Fundación para Combatir la Leucemia).

**References**

ALLISON, A.C., HARRINGTON, J. & BIRBECK, M. (1966). An examination of the cytotoxic effect of silica on macrophages. *J. Exp. Med.*, **124**, 141.

ATTIA, M.A.M. & WEISS, D.W. (1966). Immunology of spontaneous mammary carcinomas in mice. V. Acquired tumour resistance and enhancement in strain A mice infected with mammary tumour virus. *Cancer Res.*, **26**, 1787.

BASHFORD, E. (Ed.). (1908). *Third Scientific Report on the Investigation of the Imperial Cancer Research Fund.* London: Taylor and Francis, p. 262.

BIOZZI, G., BENACERRAF, B. & HALPERN, B.N. (1953). Quantitative study of the granulopoietic activity of the reticuloendothelial system. II. A study of the kinetics of the granulopoietic activity of the R.E.S. in relation to the dose of carbon injected. *Br. J. Exp. Pathol.*, **34**, 441.

CRILE, G. JR. & DEODHAR, S.D. (1971). Role of preoperative irradiation in prolonging concomitant immunity and preventing metastases in mice. *Cancer*, **27**, 629.

DECKERS, P.J., DAVIS, R.C., PARKER, G.A. & MANNICK, J.A. (1973). The effect of tumour size on concomitant tumour immunity. *Cancer Res.*, **33**, 33.

DJEU, J., HEINBAUGH, J., VIERA, W., HOLDEN, H. & HERBERMAN, R. (1979). The effect of immunopharmacological agents on mouse natural cell-mediated cytotoxicity and on its augmentation by Poly I: C. *Immunopharmacology*, **1**, 231.

EHRLICH, P. (1906). Experimentelle Carcinomstudien an Mäusen. *Arb. Inst. Exp. Ther. Frankfurt*, **1**, 65.

GORELIK, E. (1982). Antimetastatic concomitant immunity. In: *Tumour Invasion and Metastasis*. (Eds. Liotta & Hart), The Hague: Martinus Nijhoff Publishers, p. 113.

GORELIK, E. (1983*a*). Resistance of tumour-bearing mice to a second challenge. *Cancer Res.*, **43**, 138.

GORELIK, E. (1983*b*). Concomitant tumour immunity and the resistance to a second tumour challenge. *Adv. Cancer Res.*, **39**, 71.

GORELIK, E., SEGAL, S. & FELDMAN, M. (1981). On the mechanism of tumour "concomitant immunity". *Int. J. Cancer*, **27**, 847.

HEWITT, H.B. (1978). The choice of animal tumours for experimental studies of cancer therapy. *Adv. Cancer Res.* **27**, 149.

HEWITT, H.B., BLAKE, E.R. & WALDER, A.S. (1976). A critique of the evidence for active host defence against cancer, based on personal studies of 27 murine tumours of spontaneous origin. *Br. J. Cancer*, **33**, 241.

KEARNEY, R. & NELSON, D.S. (1973). Concomitant immunity to syngeneic methylcholanthrene-induced tumours in mice. Occurrence and specificity of concomitant immunity. *Aust. J. Exp. Biol. Med. Sci.*, **51**, 723.

LEVY, M.H. & WHEELOCK, E.F. (1975). Effects of intravenous silica on immune and non-immune functions of the murine host. *J. Immunol.*, **115**, 41.

MALENICA, B. & MILAS, L. (1979). Effect of tumour immunogenicity on the development of concomitant immunity. *Period. Biol.*, **81**, 331.

MIDDLE, J.G. & EMBLETON, M.J. (1981). Naturally arising tumours of the inbred WAB/Not rat strain. II Immunogenicity of transplanted tumours. *J. Natl Cancer Inst.*, **67**, 637.

NORTH, R.J., DYE, E.S. & MILLS, C.D. (1982). T cell-mediated negative regulation of concomitant antitumour immunity as an obstacle to adoptive immunotherapy of established tumours. In: *The Potential Role of T Cells in Cancer Therapy*. (Eds. Fefer & Goldstein), New York: Raven Press, p. 65.

NORTH, R. & KIRSTEIN, D. (1977). T-cell mediated concomitant immunity to syngeneic tumours. I Activated macrophages as the expressors of non-specific immunity to unrelated tumours and bacterial parasites. *J. Exp. Med.*, **145**, 275.

PASQUALINI, C.D., SEN, L., SAAL, F., SCHWARTZ, L. & TKACZEVSKI, L.D. DE (1973). Tumour development in mice bearing a plastic cylinder and inoculated with human neoplastic cells. *J. Natl Cancer Inst.*, **51**, 283.

ROFFO, A.H. (1914). *Cancer Experimental*. Buenos Aires: Las Ciencias.

SOUTHAM, C.M. (1968). Co-existence of allogeneic tumour growth and homograft immunity in man. *Eur. J. Cancer*, **4**, 507.

SOUTHAM, C.M. & BRUNSCHWIG, A. (1961). Quantitative studies of autotransplantation of human cancer. *Cancer*, **14**, 971.

VAAGE, J. (1971). Concomitant immunity and specific depression of immunity by residual or reinjected syngeneic tumour tissue. *Cancer Res.*, **31**, 1655.

WINN, H.J. (1961). Immune mechanisms in homotransplantation II. Quantitative assay of the immunological activity of lymphoid cells stimulated by tumour homograft. *J. Immunol.*, **86**, 228.

WOGLOM, W.H. (1929). Immunity to transplantable tumours. *Cancer Rev.*, **4**, 129.

YUHAS, J.M., PAZMIÑO, N.H. & WAGNER, E. (1975). Development of concomitant immunity in mice bearing the weakly immunogenic line 1 lung carcinoma. *Cancer Res.*, **35**, 237.


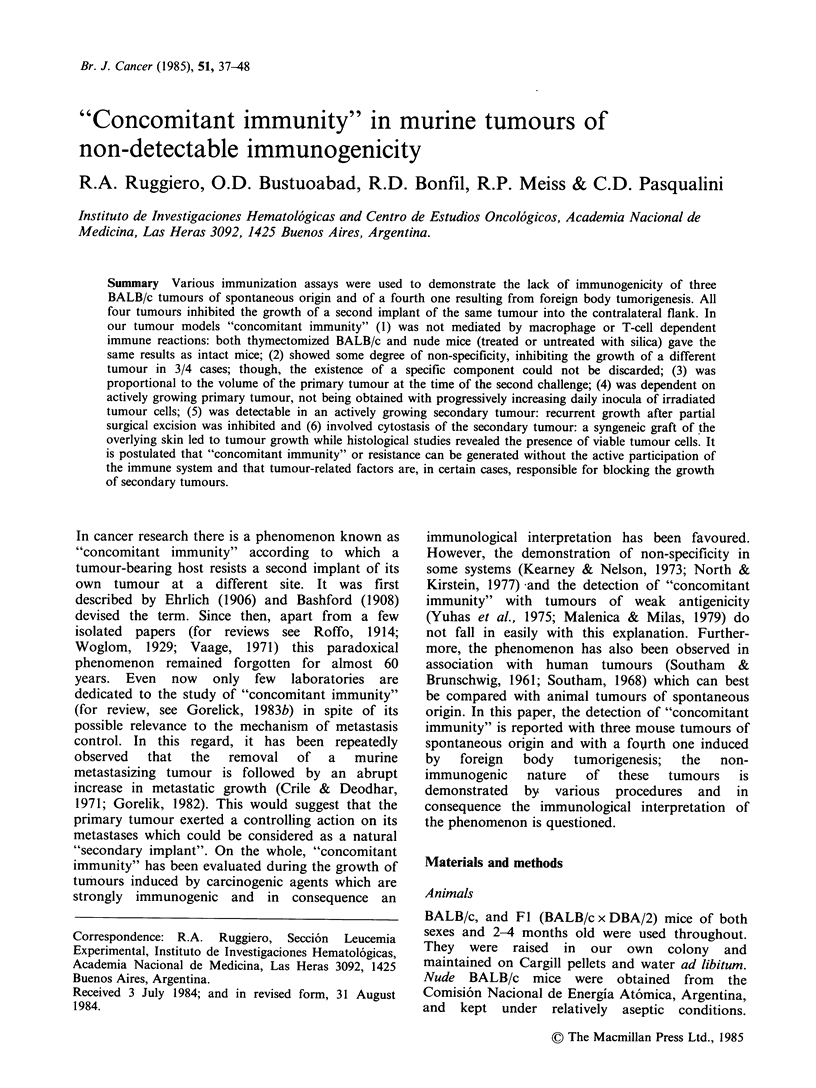

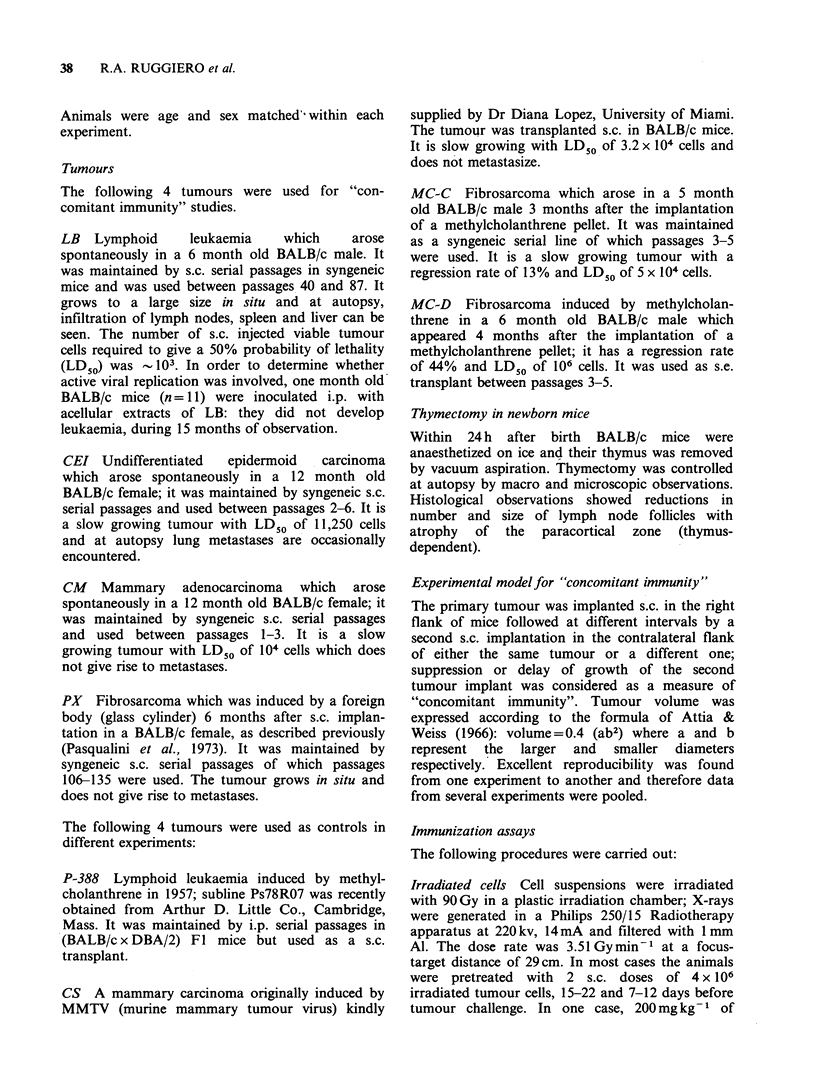

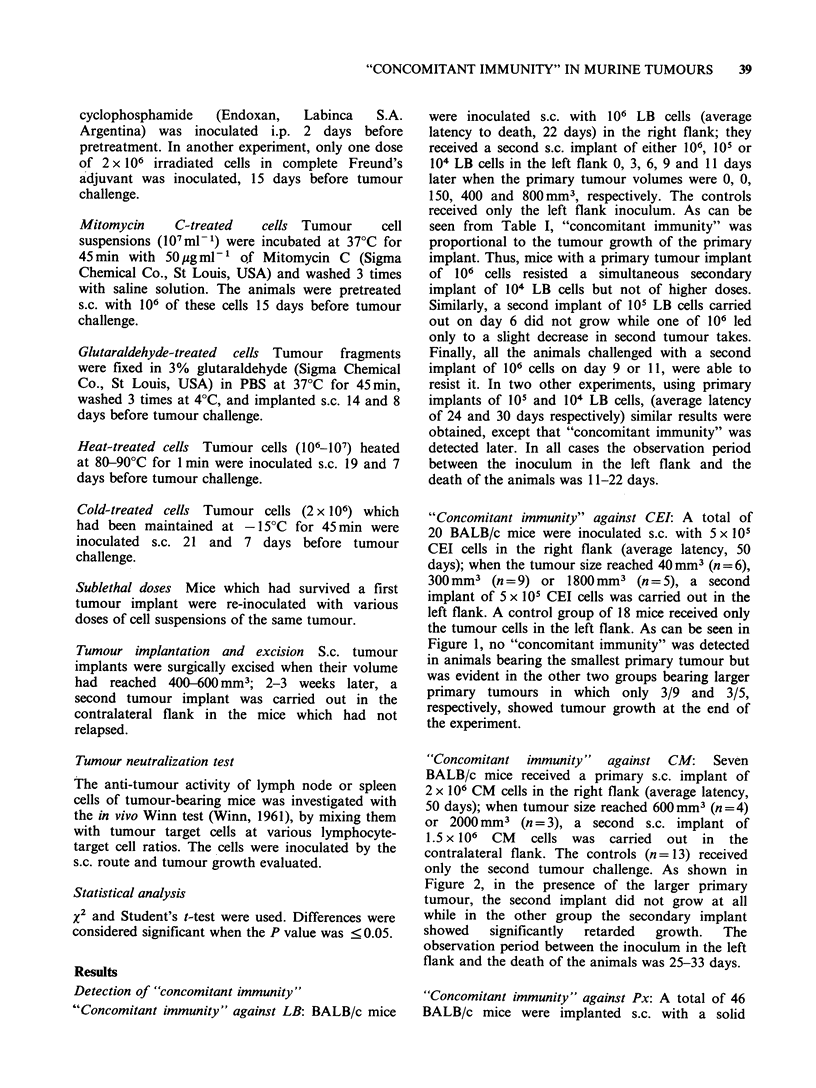

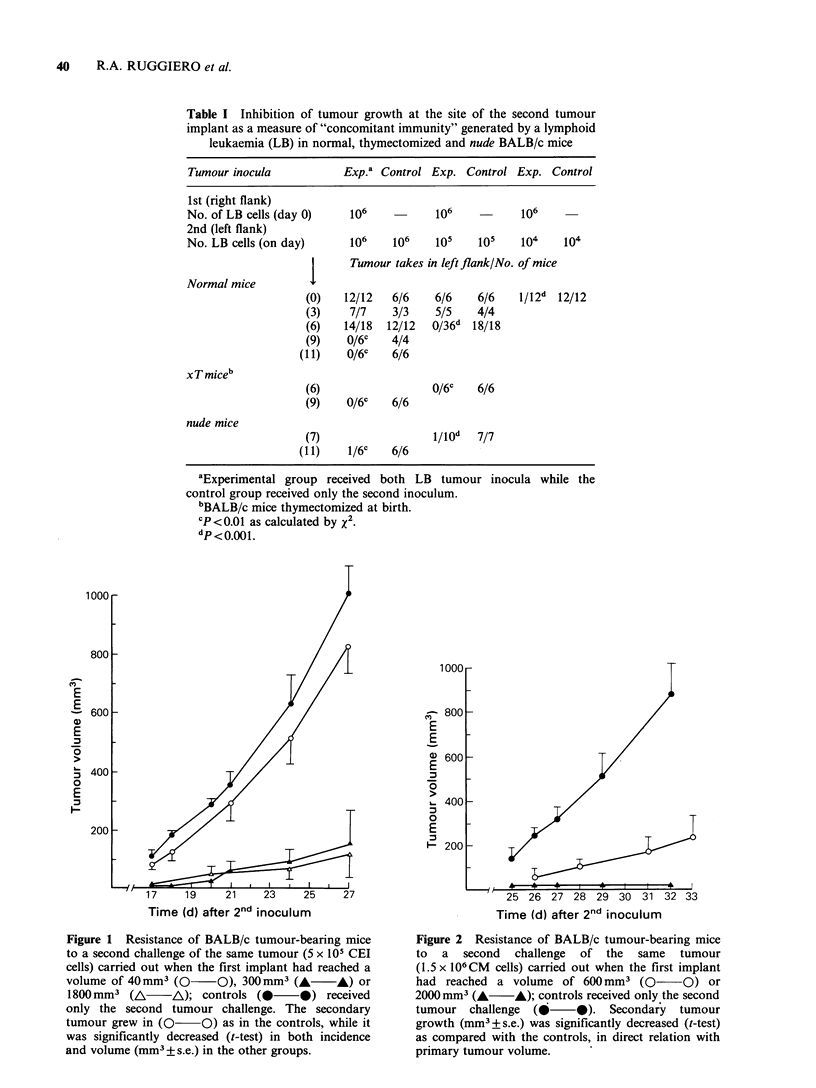

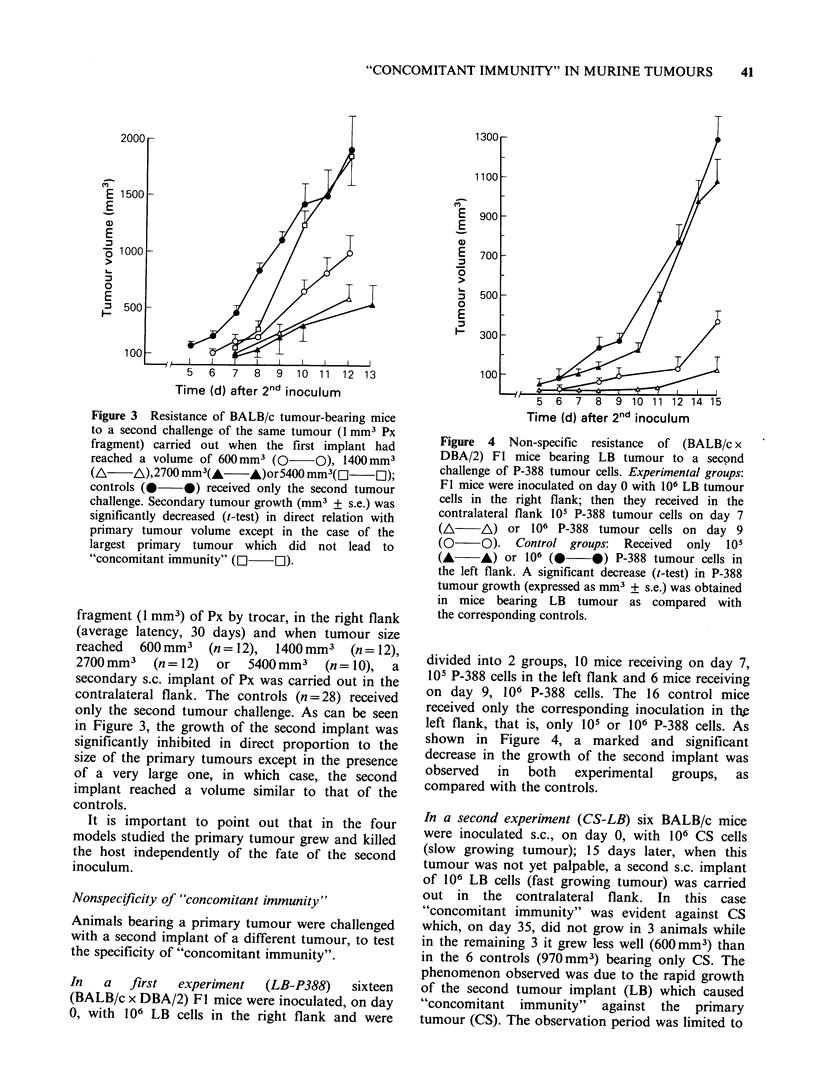

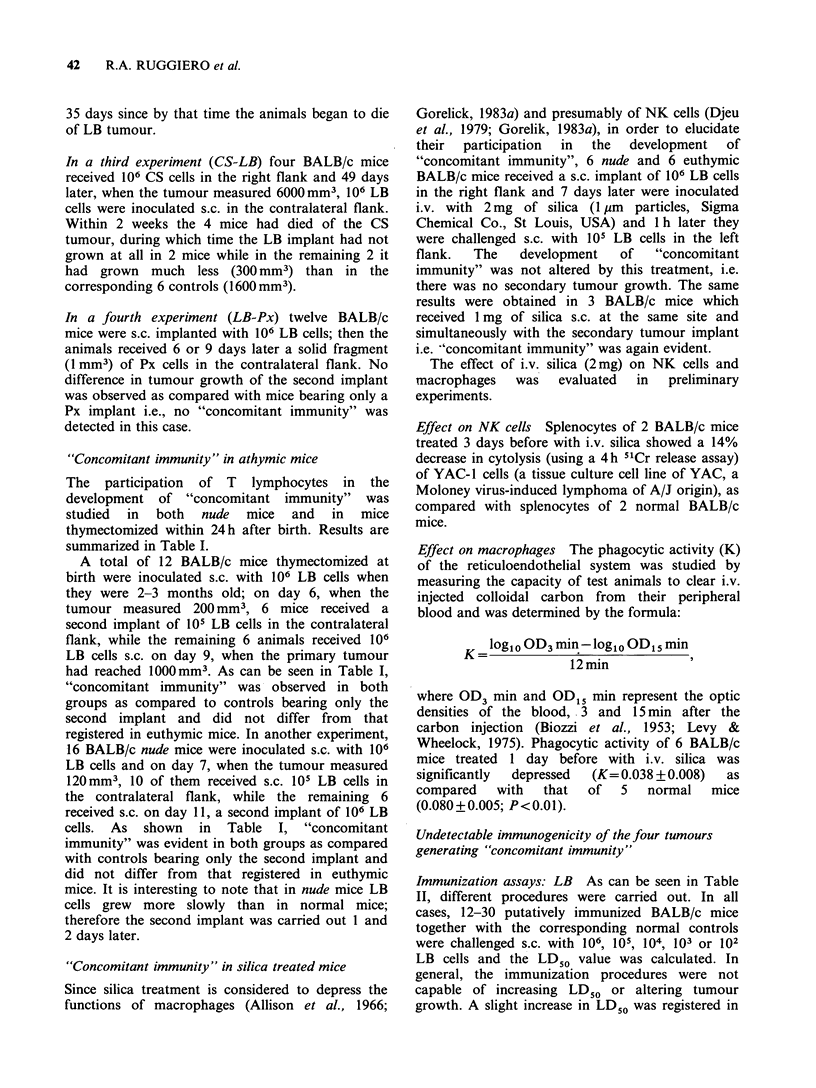

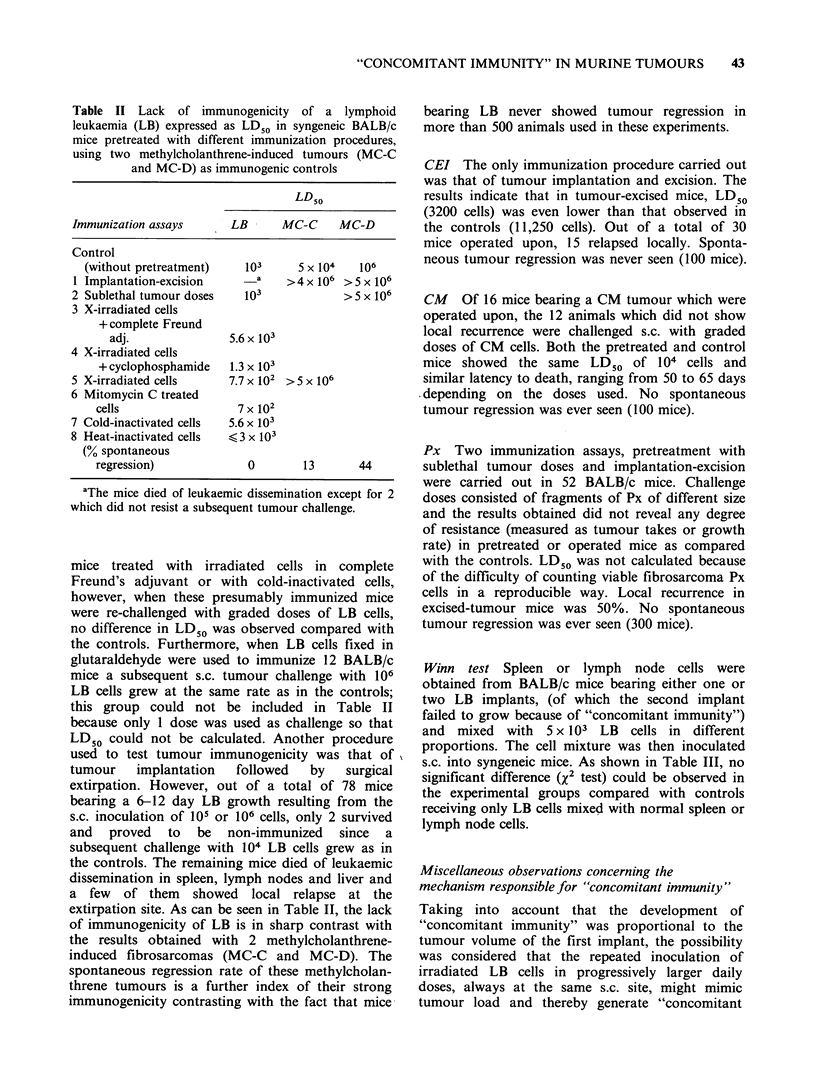

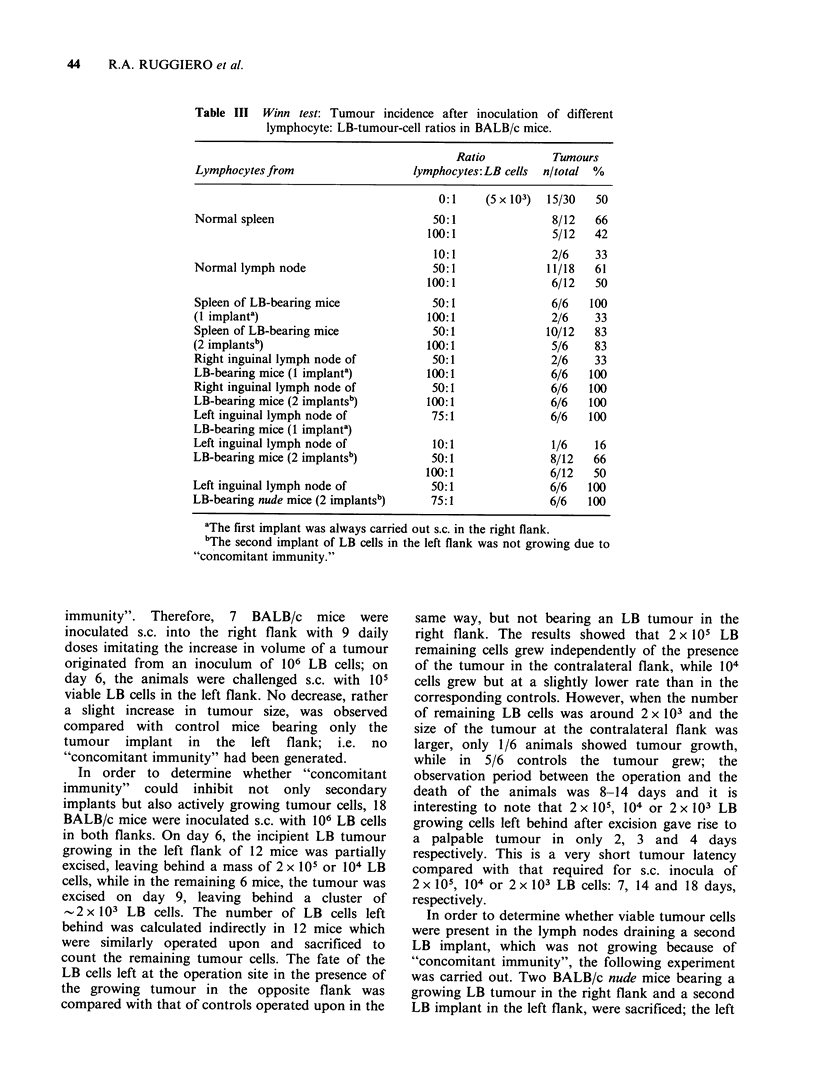

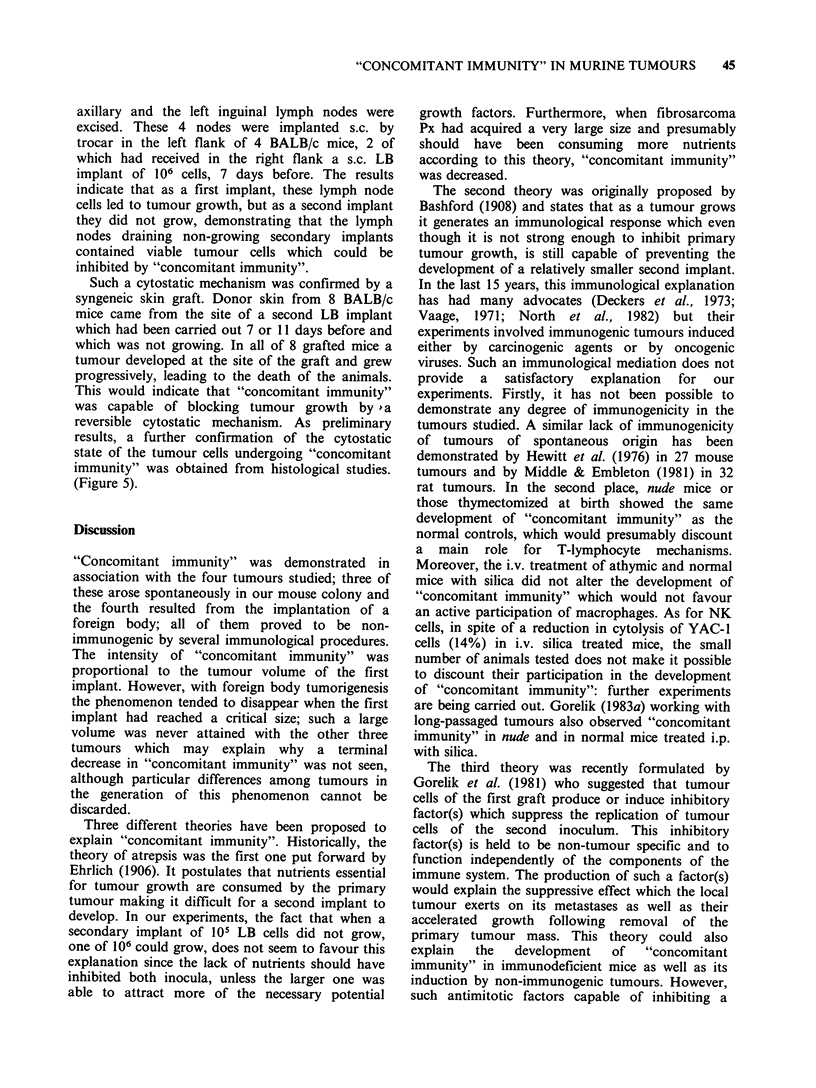

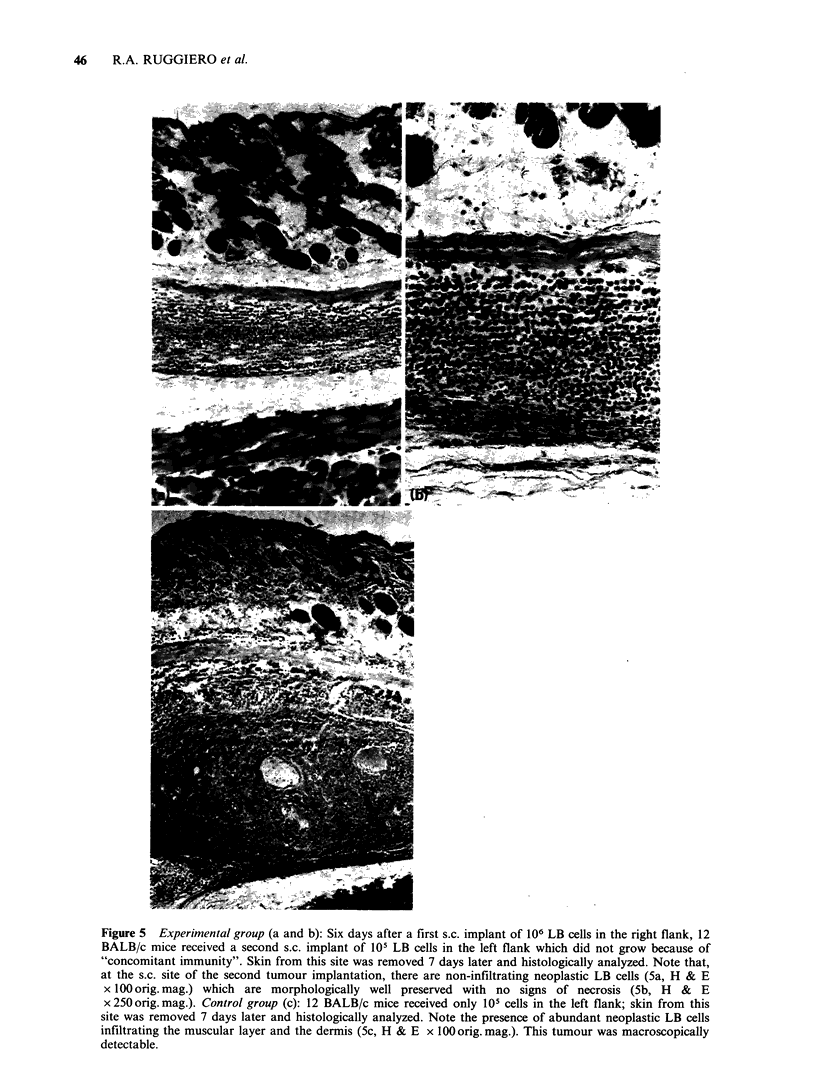

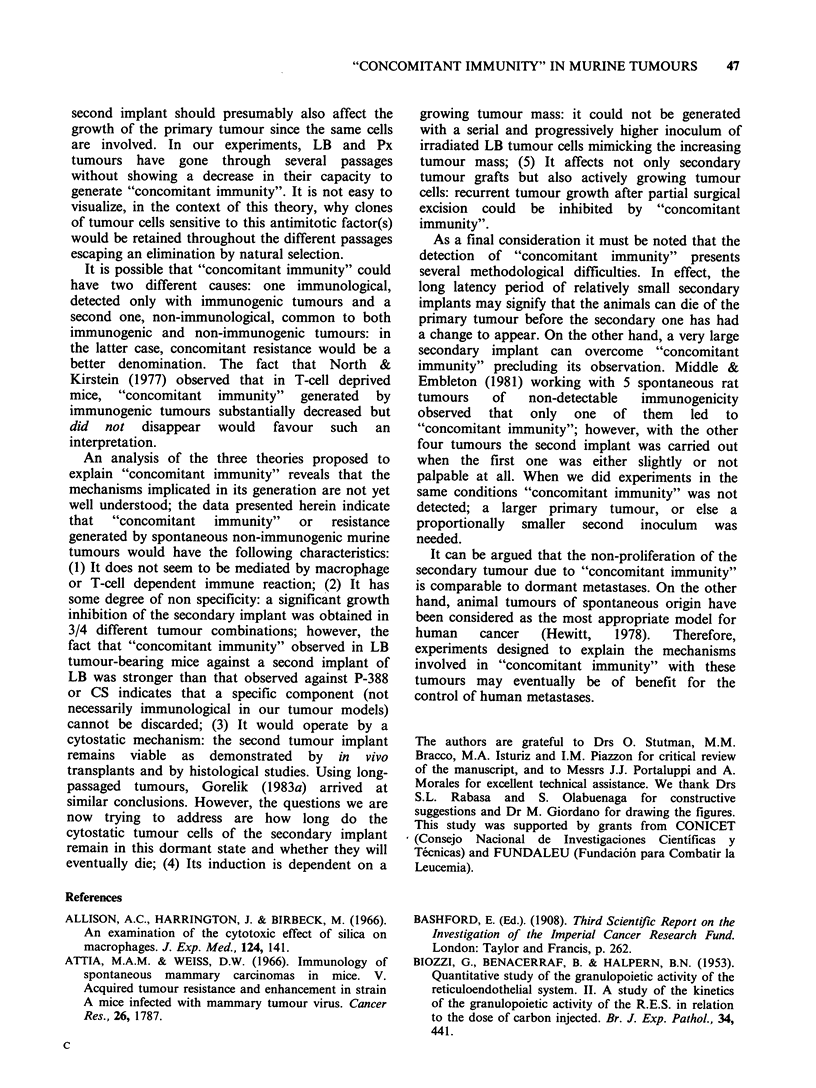

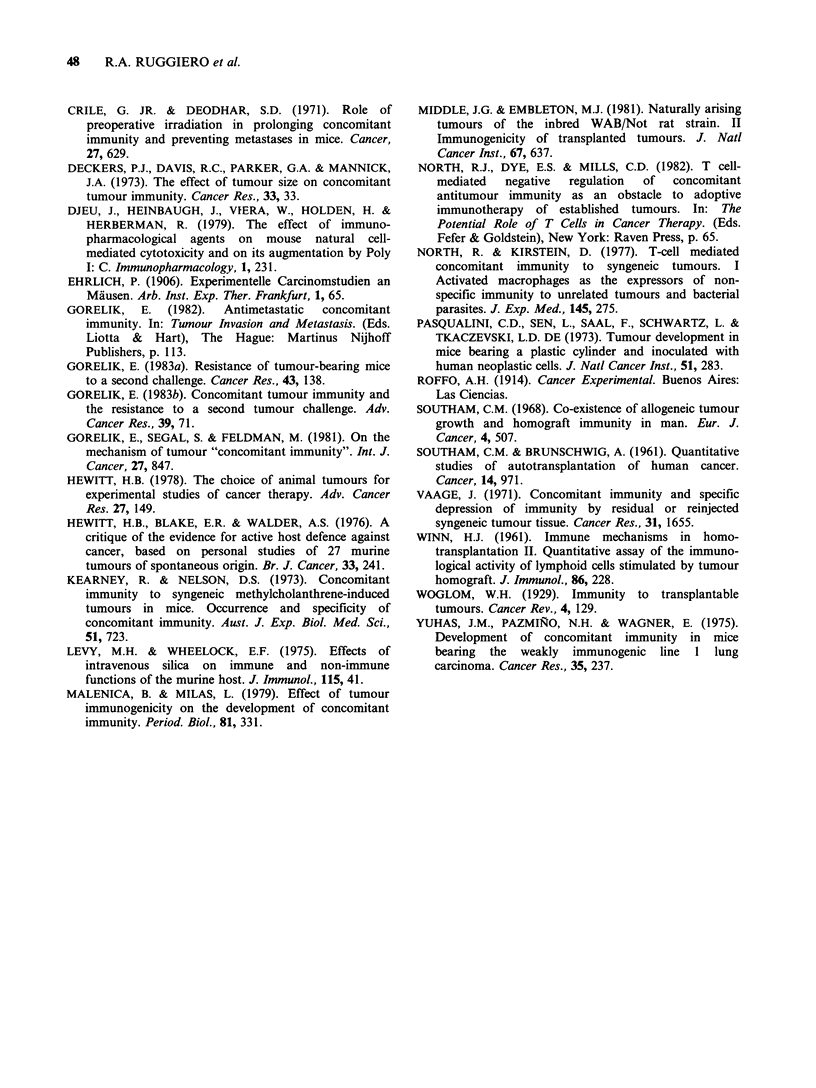


## References

[OCR_01189] Allison A. C., Harington J. S., Birbeck M. (1966). An examination of the cytotoxic effects of silica on macrophages.. J Exp Med.

[OCR_01194] Attia M. A., Weiss D. W. (1966). Immunology of spontaneous mammary carcinomas in mice. V. Acquired tumor resistance and enhancement in strain A mice infected with mammary tumor virus.. Cancer Res.

[OCR_01206] BIOZZI G., BENACERRAF B., HALPERN B. N. (1953). Quantitative study of the granulopectic activity of the reticulo-endothelial system. II. A study of the kinetics of the R. E. S. in relation to the dose of carbon injected; relationship between the weight of the organs and their activity.. Br J Exp Pathol.

[OCR_01218] Crile G., Deodhar S. D. (1971). Role of preoperative irradiation in prolonging concomitant immunity and preventing metastasis in mice.. Cancer.

[OCR_01224] Deckers P. J., Davis R. C., Parker G. A., Mannick J. A. (1973). The effect of tumor size on concomitant tumor immunity.. Cancer Res.

[OCR_01229] Djeu J. Y., Heinbaugh J. A., Vieira W. D., Holden H. T., Herberman R. B. (1979). The effect of immunopharmacological agents on mouse natural cell-mediated cytotoxicity and on its augmentation by poly I:C.. Immunopharmacology.

[OCR_01250] Gorelik E. (1983). Concomitant tumor immunity and the resistance to a second tumor challenge.. Adv Cancer Res.

[OCR_01246] Gorelik E. (1983). Resistance of tumor-bearing mice to a second tumor challenge.. Cancer Res.

[OCR_01255] Gorelik E., Segal S., Feldman M. (1981). On the mechanism of tumor "concomitant immunity".. Int J Cancer.

[OCR_01265] Hewitt H. B., Blake E. R., Walder A. S. (1976). A critique of the evidence for active host defence against cancer, based on personal studies of 27 murine tumours of spontaneous origin.. Br J Cancer.

[OCR_01260] Hewitt H. B. (1978). The choice of animal tumors for experimental studies of cancer therapy.. Adv Cancer Res.

[OCR_01271] Kearney R., Nelson D. S. (1973). Concomitant immunity to syngeneic methylcholanthrene-induced tumours in mice. Occurrence and specificity of concomitant immunity.. Aust J Exp Biol Med Sci.

[OCR_01278] Levy M. H., Wheelock E. F. (1975). Effects of intravenous silica on immune and non-immune functions of the murine host.. J Immunol.

[OCR_01288] Middle J. G., Embleton M. J. (1981). Naturally arising tumors of the inbred WAB/Not rat strain. II. Immunogenicity of transplanted tumors.. J Natl Cancer Inst.

[OCR_01302] North R. J., Kirstein D. P. (1977). T-cell-mediated concomitant immunity to syngeneic tumors. I. Activated macrophages as the expressors of nonspecific immunity to unrelated tumors and bacterial parasites.. J Exp Med.

[OCR_01309] Pasqualini C. D., Sen L., Saal F., Schwartz L., Tkaczevski L. Z. (1973). Tumor development in mice bearing a plastic cylinder and inoculated with human neoplastic cells.. J Natl Cancer Inst.

[OCR_01319] Southam C. M. (1968). Co-existence of allogeneic tumour growth and homograft immunity in man.. Eur J Cancer.

[OCR_01329] Vaage J. (1971). Concomitant immunity and specific depression of immunity by residual or reinjected syngeneic tumor tissue.. Cancer Res.

[OCR_01334] WINN H. J. (1961). Immune mechanisms in homotransplantation. II. Quantitative assay of the immunologic activity of lymphoid cells stimulated by tumor homografts.. J Immunol.

[OCR_01344] Yuhas J. M., Pazmiño N. H., Wagner E. (1975). Development of concomitant immunity in mice bearing the weakly immunogenic line 1 lung carcinoma.. Cancer Res.

